# ADAPTING MINDFULNESS-BASED COUNSELING FOR THE TELEPHONE: A PILOT STUDY FOR CAREGIVERS AND VETERANS WITH DEMENTIA

**DOI:** 10.1093/geroni/igz038.2074

**Published:** 2019-11-08

**Authors:** Michelle M Hilgeman, Phoebe R Block, Teddy K Bishop, Kimberly Alexander, A Lynn Snow

**Affiliations:** 1 Tuscaloosa VA Medical Center, Tuscaloosa, Alabama, United States; 2 The University of Alabama, Tuscaloosa, Alabama, United States; 3 Tuscaloosa VA Medical Center, The University of Alabama, Tuscaloosa, Alabama, United States

## Abstract

Optimizing Dementia Care in Veterans with Dementia is a randomized, controlled, pilot study examining outcomes for Veterans and their caregivers at 6- and 12-months for two telephone-based interventions: a) Benjamin Rose Institute’s (BRI) Care Consultation (CC), and b) CC + Counseling (CC+C). Counseling modules are integrated into the existing BRI CC framework using guided mindfulness-based skill-building exercises on various content domains (e.g., grief, identity, intimacy, stress management). Sixty-four caregivers and 47 Veterans (M = 74.3 years, MOCA Score M = 15.5) have been randomized in this ongoing pilot study. Caregivers are 91% female, 32% Black/African American, and 72% spouses. Preliminary implementation and 6-month outcome data is discussed (e.g., reaction to behavioral distress, mindfulness, depression, quality of life) using within-group paired samples t-tests for the 32 dyads randomized to CC+C. Lessons learned include strategies for adapting mindfulness-based approaches over the telephone to enhance access for Veterans and caregivers across geographic regions.

